# The continuous search for new port governance models: experiences from a developing country

**DOI:** 10.1186/s41072-023-00139-8

**Published:** 2023-04-11

**Authors:** Torben Andersen, Jonas Aryee, George Acheampong, Annette Skovsted Hansen

**Affiliations:** 1 Aarhus University, Aarhus, Denmark; 2 Regional Maritime University, Tema, Ghana; 3 University of Ghana, Legon, Ghana

**Keywords:** Port governance, Stakeholder theory, Inclusive port development

## Abstract

This paper bridges port governance and stakeholder theory to contribute to new understandings of changed stakeholder relations due to the building of new container terminals. The case of the newly inaugurated very large high-tech container terminal commissioned to and developed and operated by Meridian Port Services (MPS), in the Port of Tema, Ghana, provides the empirical foundation for investigating the new stakeholder engagement. Through focus groups, descriptive statistics, and a series of qualitative and open-ended interviews carried out in structured stakeholder events, the paper aims to deliver new knowledge relevant to the many hybrid port governance systems seen today. Moreover, it wants to inform authorities and companies about the implications of different strategic choices and how changes progress. The study shows that despite shared goals of efficiency, all parties involved have had major difficulties in finding a new port governance model, which they all consider fair and which can contribute to a continuously economically sustainable port management practice.

## Introduction

The role of ports as growth tools for development in many emerging countries has been highlighted in extant literature (Cong et al. 2020; Ma et al. 2021). However, what governance models to adopt remains problematic. This study sheds light on this issue by drawing on the empirical evidence from the concession and development of a container terminal at the Port of Tema in Ghana by the Meridian Port Services (MPS) consortium. We explore how various stakeholders develop and build an immense leap in port infrastructure expansion but fail to find an operating model that all actors consider fair. We aim to show the implications of different strategic choices and how the changes progress. The need for the port infrastructure expansion was the estimated future throughput increase. According to the International Transport Forum (ITF) (2021), freight is estimated to increase by 2.6-fold in 2050 compared to 2015. Most of this continuous growth will be carried by container ships and go through container terminals, placing various demands on ports and port governance worldwide.

Within the last 10–15 years, we have seen an increase in the variety of governance models due to national-level port governance and management reforms. Most studies have analysed these reforms in the Americas, Europe, the Middle East, and Asia. However, changes are now increasing on the African continent, which offers opportunities for developing new understandings of port governance models seen from both an empirical and a theoretical point of view. Most studies have focused on global maritime centres in Asia and Europe—e.g., the privatisation of United Kingdom ports, structural changes in Italian and Greek port systems, the decentralisation of port management in China, the major privatisations in France and Spain and the German-Dutch-Belgian port system (Dombois and Koutsoutos 2007). Our study investigates how port governance and management systems develop in new contexts, in this case, Tema Port in Ghana, West Africa, where the Ghanaian government chose the landlord port model in 1999, which allowed for concessions such as the MPS Terminal 2 and Terminal 3 that opened in 2004 and 2019, respectively. However, other governance models exist beyond the landlord port model, such as the private, tool, and public service ports (Brooks 2009). The differences between these models are based on factors such as the type of service provider (public, private, or mixed), their orientation (local, regional, or global), the ownership of infrastructure, superstructure and assets, and the status of dock labour and management (World Bank 2007; Notteboom and Haralambides 2020).

Considering the African continent, there seem to be significant differences compared to other continents, not the least regarding public involvement. Therefore, will the government of Ghana maintain the landlord port model or evolve into other governance models? With service and tool ports primarily serving public interests, private ports have mostly been acting in the interest of private shareholders and landlord port authorities, trying to balance public and private goals. According to Notteboom and Haralambides (2020), the landlord model is the most common model for port administration in more than 80% of ports worldwide. Among their many responsibilities, port authorities have been the curator and the authorised managers of port land and adjacent aquatic areas to be rented out (leased) for economic profit to the private sector. Often, revenues from this activity amount to 50% of total port revenue. Consequently, changes in port governance and management are, in other words, a domain for potential stakeholder conflicting interests (De Langen 2007). Therefore, the question concerns who benefits, and what are the implications of redistributing power and responsibility among public or private, capital or labour, and local or global actors?

A large body of port economics literature has analysed how the governance model of ports can dramatically change because of far-reaching port reform and devolution programs (see Cullinane and Brooks 2006; Brooks et al. 2017; Pallis et al. 2011; Zhang et al. 2018). Out of the existing literature, the transition role of the public sector in ports has attracted particular attention. However, the same literature also highlights that many privatisation, corporatisation, and commercialisation schemes have occurred in many parts of the world in the last decade (Haralambides 2017; Notteboom and Winkelmans 2001). This has resulted in the entry of global terminal operating and logistics groups, large investment groups, and equity fund managers. In the present article, we look at how this has occurred in a specific context on the African continent.

The infusion of private investments has led to greater competition on a global scale for multinational companies, and the implications for local governments are profound. Governments claim expectations of higher productivity locally and, eventually, lower total costs, which can pass and be passed on to domestic actors like importers and exporters. Consequently, the public sector has been forced to reassess its role in the port industry in this new environment. Many countries have now implemented some form of commercialisation or corporatisation of public port authorities to deflect demands for much greater private sector involvement and safeguard the prerogatives and collective interests of the public sector. Notteboom and Haralambides (2020) describe this as a situation where extensive and lengthy processes of layering involving multiple incremental changes and adaptations can result in a gradual mutation of the role of the public sector actors.

The aim to obtain a better fit between the port governance system and the local/regional socio-economic environment seems elusive as the private entities assume more power than the public authorities, reducing their ability to provide fair treatment of all stakeholders. The argument is that even subtle and stepwise changes in port governance can have a significant longer-term impact on the functioning and performance of the port. Potentially, growth and economic prosperity are achieved at the expense of limited possibilities for public sector successive control and interference in what used to be policy matters. In this light, we chose to view the building of the new MPS terminal in the Port of Tema, which opened in June 2019, as a study of stakeholder theory and port governance in a developing country. This is also because developing countries’ public sectors are highly politicised and susceptible to influence from powerful multinationals, as illustrated by the cases reported by Weir (2021) and Kazeem (2018).

Firstly, this article focuses on the stakeholder theory’s key contributions and underlying assumptions to map the main actors involved in changing the old and developing new governance models. Secondly, we rely on the extensive material published in earlier studies, specifically addressing stakeholder theory in port governance. Then, we present our methodological setup for our two field studies in Tema, Ghana, and describe the new MPS Terminal in the Port of Tema and the difficulties the actors experienced in finding an operating system considered fair by all stakeholders. Finally, we suggest a model for more sustainable stakeholder inclusion as a way forward for MPS, the port authorities and all the other stakeholders in the old port and the new terminal in Tema, covering values, norms, and ethical elements before concluding.

## Stakeholder theory

Stakeholder theory argues that corporations should act beyond the financial interests of the few persons that constitute their shareholders to the broader social interests of all its stakeholders (Freeman and Phillips 2002). Much of the literature on stakeholder theory has, in other words, been devoted to defining and justifying the stakeholder perspective or, from an empirical perspective, proving that seeking to satisfy a broad group of stakeholders is economically justifiable. In this light, Freeman (1984) initially defined stakeholders as “…*any group or individual who is affected by or can affect the achievement of an organisation’s objectives*”. Stakeholder theory’s central management suggestion is that effectively managing relationships with a firm’s stakeholders are the primary responsibility of managers and central to shared value creation; it is a plus-sum operation.

In this paper, stakeholders refer to individuals and groups (other companies and communities) with a vested interest in the activities of the focal organisation, the Ghana Port and Habour Authority (GPHA). GPHA actions within the context of this paper refer to the changes in the governance models implemented by the GPHA. Private and public sector actors have a lot at stake when the governance systems change, and stakeholder theory, as we know it from the mid-1980s and early 1990s, has tried to map and reflect on the pros and cons of the various systems. The theoretical approach has proven its worth in mapping an increasingly complex and interconnected external and internal environment of organisations. However, the focus has been, to a significant extent, on boundaries and, thereby, on what is inside or outside the organisation, i.e., who has a legitimate right to be a stakeholder in shared decisions on governance models.

Stakeholder theory advances that organisations taking excellent care of a broad group of stakeholders, e.g., their customers, suppliers, employees, and communities, function more effectively and create more value for all in the long run. This value sustains and grows the organisation and, in some form, secures the return to the stakeholders who helped create the organisation. Emphasis on stakeholder relations and theory is both managerial and prescriptive because it deals very specifically with manager behaviour and the relationships between a firm and its constituencies. The theory also rests on a strong ethical foundation.

In stakeholder theory, firms that focus on their stakeholders often provide more value to their stakeholders than necessary. This type of behaviour, when combined with trust stemming from organisational justice and adherence to ethical principles, leads to trusting, respectful and mutually beneficial relationships with stakeholders and a high level of reciprocation. Stakeholders are likelier to share valuable information with such firms, leading to efficiency and innovation. In an earlier study, we mapped some of these thoughts (see Acheampong et al. 2022). Resources are easier to obtain because stakeholders expect a fair return. Trust in firms reduces contracting costs, and fewer features of the contracts between a firm and its stakeholders must be recorded and carefully monitored. This leads to firm growth, efficiency, flexibility and, consequently, an increased ability to make and implement plans. These types of firms appear to function better and seem less prone to become victims of negative stakeholder actions such as walkouts, boycotts, legal suits and bad press. Consequently, their securities may be seen as less risky and more valuable to investors.

Finally, treating all stakeholders well is central to stakeholder theory (Zaucha and Kreiner 2021). Although there is no consensus on what it means to treat stakeholders well, certain principles exist regarding the treatment of stakeholders that are widely accepted among those who advance the theory. These principles rely primarily on ethical thinking, which means, in part, that core rules that are based on socially accepted norms of behaviour, e.g., lying is wrong, determine the evaluation of a firm’s actions concerning its stakeholders. From a stakeholder perspective, firm behaviour may also be judged based on outcomes. Firms are expected to produce favourable results based on achieving morally important goals. For instance, a for-profit corporation is expected to create goods and/or services that satisfy consumer needs, thus providing the means for employees to take care of their physical needs and families, help the communities in which they operate, and offer fair returns to shareholders, among other things.

### Stakeholder theory in port governance

Stakeholder theory’s popularity in port management studies has been very profound (e.g., Ha et al. 2017; Kothuis and Slinger 2018; Dooms 2018). These studies include a wide variety of internal stakeholders, e.g., those actors who are directly part of the port administration organisation, shareholders, managers, employees, unions, and external stakeholders. The latter group includes actors ranging from economic players directly investing in the port area (e.g., concessionaries, freight forwarders, carriers and port service providers) to firms or institutions located in the foreland or hinterland (e.g., shippers and multimodal transport operators), cruise and ferry passengers, public policy stakeholders and regulators, as well as local community and societal groups of interest. Port stakeholders constitute groups and individuals interested in the activities and outcomes of a port as an organisation and on whom the port relies for achieving its objectives. For example, customers of the various actors in the complex port value chain constitute one group of stakeholders, e.g., they have an economic stake. Suppliers and employees are examples of other stakeholders with an economic stake in ports. Stakeholders might also have an equity stake in the firm, including the port authorities and the two large multinational companies, such as MPS consortium shareholders. In addition, stakeholders may simply be interested in what the firm does because it influences them somehow, even if it is not a direct market effect.

Special interest groups, for instance, try to influence firm decisions in conformance with their agendas. Stakeholder coalitions often form around particular issues because stakeholder interests tend to be interconnected. Various stakeholder groups receive any organisational action favourably or unfavourably. The influencer stakeholder highlights a critical point: just because a stakeholder is interested in the organisation does not necessarily mean that the organisation is particularly interested in that stakeholder. In other words, there is no universally accepted definition of who merits classification as a legitimate stakeholder from the organisation’s perspective. Managers generally consider stakeholders salient to their port organisation if they have power and legitimacy (Zaucha and Kreiner 2021). As Freeman et al. (2010) states, “[*b*]*usiness works because the interests of all the stakeholders can be satisfied over time (and not that some groups always have priority over others)*”. In other words, stakeholders have power if they possess critical resources which the firm needs or can influence outcomes through political, coercive, or other means. Legitimacy pertains to cultural and societal norms. For instance, a stakeholder may be considered salient to a manager because doing so is considered desirable, proper, or appropriate given the circumstances. In addition to power and legitimacy, a stakeholder could become important in urgent situations, where urgency means that a particular stakeholder’s claim is time-sensitive or critical to the stakeholder.

Another way to determine which stakeholders should receive primary attention is the principle of fairness. This principle suggests that the organisation’s legitimate stakeholders should include voluntarily accepted resources. In an earlier study (see Acheampong et al. 2022), we reported the primary stakeholders’ legitimacy, including employees, customers, financiers, suppliers and local communities. Their integral link to the value-creating processes of the organisation makes them primary. Secondary stakeholders can dramatically influence an organisation but typically are not a part of the firm’s operating core. Examples of secondary stakeholders include the government, the media, special interest groups, consumer advocate groups and competitors.

Stakeholder theory received criticism early in its development from people, who claimed that it advances the position that all stakeholders should have equal standing with the firm. While it may be true that stakeholder theory advocates moral and just treatment of all a firm’s stakeholders, it does not argue that all stakeholders are equal. This is especially pertinent concerning the resources an organisation devotes to serving particular stakeholders and the value it allocates to returns. Fairness would suggest that more value and attention should be allocated to stakeholders central to the organisation’s objectives and who contribute the most to the firm’s value-creation processes (see also Notteboom and Winkelmans 2002).

Many port governance studies have analysed the shifts, particularly in public sector institutions. According to Notteboom and Haralambides (2020), port authorities in many countries have taken the role of a landlord, where the task is to optimise the use of its domain by (i) earmarking port areas for specific uses, (ii) awarding concessions and authorisations to a carefully selected ‘mix’ of companies, and (iii) adopting an appropriate pricing system. Despite its alleged intention to introduce more private sector operations in port administration, the landlord model is often the most bureaucratic (layered) model, given that the port authority is summoned to manage an infrastructure that belongs to the state. In fact, in many countries worldwide, the port authority is a landlord ‘on paper’ only and no more than a concessionaire, similar to ports that lease their managed areas. In many instances, the port authority has relatively limited autonomy in setting concession prices, port operator authorisation fees, wharfage charges and other dues. At the same time, it is responsible for turning out a surplus at the end of the year. This often creates what Notteboom and Haralambides (2020, p. 234) call a hopeless situation of ‘responsibility without authority’.

Thus, it has been argued that in developing a more modern port management model, national authorities should play a more proactive role in facilitating and coordinating stakeholders in logistics networks and developing the necessary competencies to succeed in highly competitive markets (Notteboom and Winkelmans 2001; Comtois and Slack 2003; Van Der Lugt and De Langen 2007), perhaps even by adopting a more entrepreneurial role (Verhoeven 2010). In addition, port authorities have also been encouraged to add a functional role as cluster managers (De Langen 2004) and community managers (Chlomoudis et al. 2003) to solve collective action problems in and around the port domain.

Instead of forcing formal (regulatory) change, the relevant stakeholders in modern port governance might stretch existing institutions and institutional arrangements through deliberate action and flexible interpretation via conversion, layering, and stretching (see Notteboom et al. 2013). As part of the soft values discussion, port authorities in many countries have attached great importance to the role of transparency and disclosure as tools in stakeholder relations management and image building in port management performance (see, for instance, Notteboom et al. (2015) on practices of the port of Rotterdam; the extensive analysis of the levels and standards of transparency in the governance of ports by Brooks et al. (2020) or the growth of sustainability reporting by port authorities in Geerts and Dooms (2017)).

Despite the renewed academic interest in transparency and disclosure, the literature may not have captured the issues related to these welcome initiatives in port management and practice. For example, ports and their decisions are often under the scrutiny and approval of traditional supervisory bodies, which usually comprise a representative group of port stakeholders (i.e., city, provincial or regional administrations; labour unions; concessionaires; railways; chambers of commerce and industry; carriers and their agents). In addition to safeguarding and promoting the interests of the port, these people may have personal or corporate interests (Zaucha and Kreiner 2021).

Therefore, indiscriminately disclosing information to other stakeholders, or even worse, the public, on ‘sensitive’ matters such as cost breakdowns, things that no commercial entity would ever disclose even to its shareholders, might be counterproductive to the long-term well-being of the port. In an increasing number of ports worldwide, the most significant part of the documentation produced by the port authority is, by law, uploaded to the organisation’s website (Geerts and Dooms 2017). Such documentation, among other information, includes executive decisions and tenders, qualified suppliers, concessions and authorisations, maintenance plans, technical department designs, budgets and much more. We still have to see whether this is also the case in the emerging economies in Africa, where political interests may be stronger and more subtle. With limited disclosure, the question remains what are the requirements for maximising the economic benefits to the broader port community and its stakeholders, including those of the host city. However, as ports operate to meet increasingly commercial criteria and in competition with other ports, an important question arises: In achieving a *level playing field* among competing ports in economically interdependent geographic areas, should authorisation allowances be included in port prices, which allegedly aim, as they should, at the recovery of port investment costs (Notteboom and Haralambides 2020)?

## The study context—Port of Tema

**Fig. 1:**
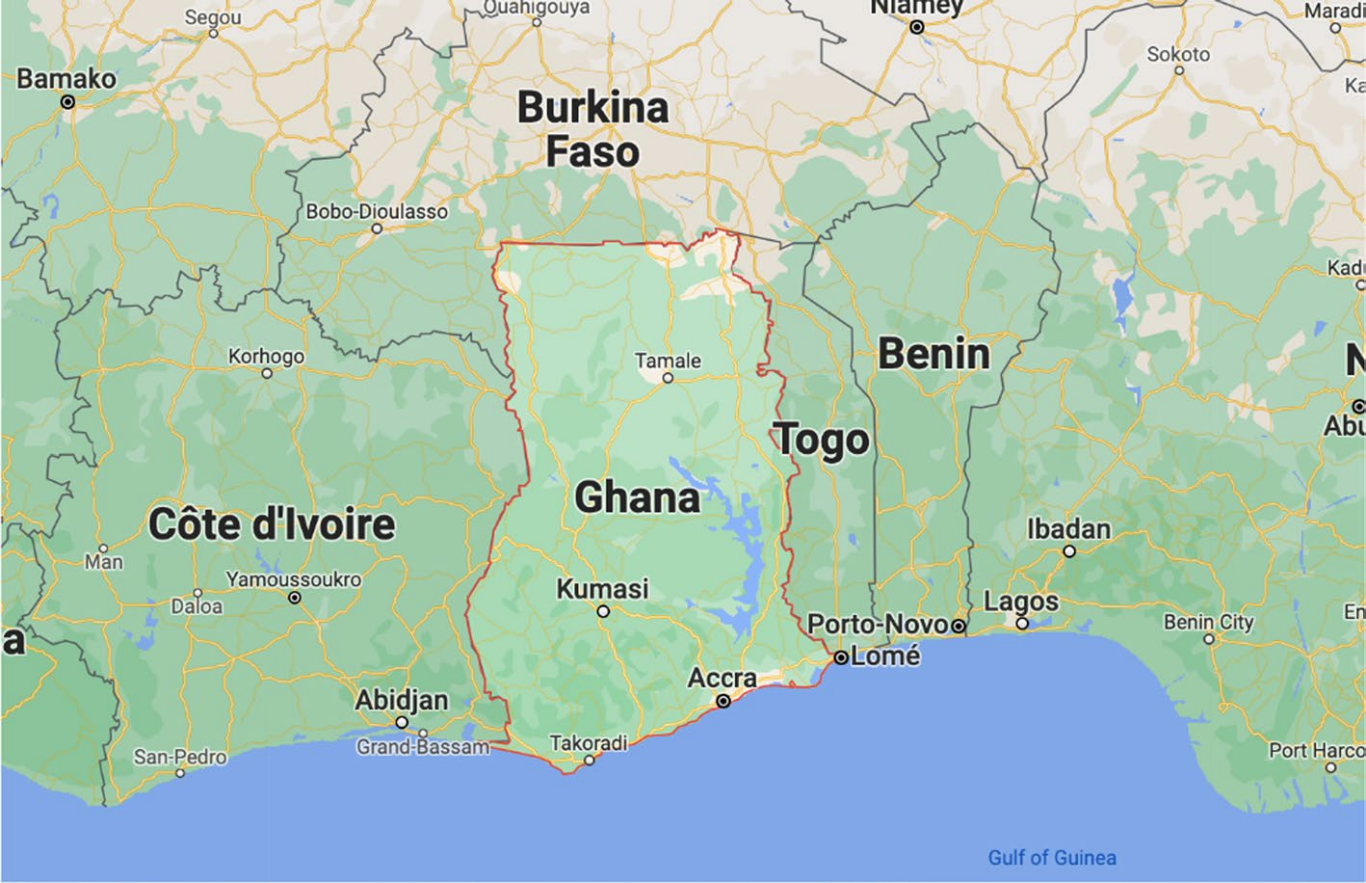
Local geographical overview—the competitive situation. *Source*: Google Maps (https://www.google.com/maps)

The Port of Tema is located about 30 km east of Ghana’s capital Accra (see Fig. 1), and was commissioned in 1962; Ghana is among Africa’s most politically stable countries. According to the World Bank, it handles most container freight in West African countries. At the same time, the port provides access to the world’s oceans for nearby landlocked countries such as Burkina Faso, Mali, and Niger.

Neighbouring ports in the region are Abidjan, 269 nautical miles (NM) away, Lomé, 83 NM away, Lagos, 220 NM away and Ghana’s other main port, Takoradi, 111 NM away. Therefore, the relatively short distance creates a potentially highly competitive environment for the handful of countries placed in the Gulf of Guinea concerning investments of global shipping lines in port facilities. However, the expected growth with the new MPS container terminal makes the Port of Tema particularly interesting. The port is set to become a hub for port traffic in West Africa, where the largest container ships can moor and ship containers via smaller container ships to nearby ports. While the Port of Tema handled 840,000 containers in 2018, the port today can handle more than 2 million containers annually using the new terminal. Table 1 illustrates the figures from the latest decade on Port of Tema performance, including data during the Covid-19 period, after the finish of the new MPS terminal. The new MPS terminal is a modern, highly automated self-service port from entry, control, and direction to exit, with advanced information technology (IT) and biometric scanning and routing tickets for truckers.

**Table 1:** The Port of Tema performance, 2010–2020

Year	Vessel call (units)	Total cargo traffic	Export	Import	Transit	Transhipment	Container traffic
		Tonnes					Teus
2000	1163	6,219,517	910,779	5,083,439	144,973	17,715	166,963
2001	1169	6,314,968	932,931	5,013,007	261,251	38,165	178,342
2002	1272	6,841,481	821,042	5,186,690	627,773	151,233	223,377
2003	1172	7,391,268	809,589	5,490,893	885,093	138,520	305,868
2004	1381	8,447,655	1,071,006	6,403,422	764,123	71,082	342,882
2005	1643	9,249,977	1,182,469	6,936,688	875,325	155,815	392,761
2006	1994	8,046,838	955,084	5,675,027	887,589	339,841	425,408
2007	1672	8,378,682	1,099,094	6,120,5837	843,656	119,209	489,147
2008	1568	8,727,049	1,305,451	6,259,412	864,307	195,326	555,009
2009	1634	7,406,490	981,075	5,694,280	509,124	192,565	525,694
2010	1787	8,696,951	1,154,826	6,423,488	447,071	236,615	590,147
2011	1667	10,748,943	1,532,139	8,431,531	614,078	171,195	756,899
2012	1521	11,468,962	1,477,390	9,383,462	530,457	50,403	824,238
2013	1553	12,180,615	1,493,956	10,014,243	620,668	51,748	841,989
2014	1504	11,126,355	1,463,273	8,922,550	577,227	163,305	732,382
2015	1514	12,145,496	1,303,090	10,043,146	722,508	76,752	782,502
2016	1521	13,414,784	1,633,036	10,890,084	862,377	29,287	893,841
2017	1543	14,045,787	1,646,253	11,327,502	1,043,771	3064	956,374
2018	1520	16,594,685	2,344,529	12,983,358	1,751,129	15,669	1,007,065
2019	1464	17,316,276	2,524,434	13,484,666	1,262,494	44,662	1,000,926
2020	1639	18,909,586	2,458,859	14,671,968	l,454,868	323,838	1,248,726

*Source*: GPHA (2021)

**Fig. 2:**
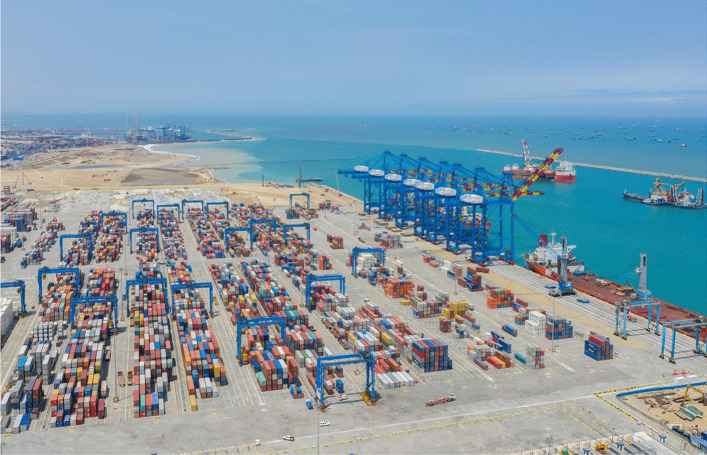
View over the new Meridian Port Services terminal. *Source*: APM Terminals (https://www.apmterminals.com/en/tema)

The MPS terminal 3 was built in three stages, while the old Tema terminals handled ongoing container traffic from 2016 to 2020. The completed terminal consists of a 1400 m long quay with 3 berths and a draught of 16 m. The container holding yard is 127 Hectares (approximately 312 acres) of land reclaimed from the sea with drainage, sewage, water, fire, electrical and IT services. It has 1400 reefer plugs, 29 e-rubber-tired gantry cranes, 4 reach stackers, 5 empty handlers, 12 MW backup power station, major facilities including administration buildings for MPS and the state agencies (authorities), a maintenance workshop, a 60-bay unstuffing shed for customs, 6 scanners, several gate facilities, a fire plant, and sewage treatment facilities (also see Fig. 2).

Now providing access to the largest container ships in the world, finishing the construction of the port and terminals on time and equipped with some of the most advanced port technology, the Port of Tema expansion has to a large degree, been labelled a success. The growth figures in vessels’ traffic, total container throughput, i.e., transit and transhipment throughput (see Table 1), are indeed a strong indicator of success. However, looking more into the Port of Tema governance, some difficulties of the long-term implications of the construction have emerged.

Regarding the construction, which started in July 2016 by the Chinese Habour and Engineering Company (CHEC) contracted by MPS, around 6000 Chinese employees were working on the site, while local Ghanaians accounted for circa 1000 employees. Such disparity indicates that the job boom for locals was relatively limited compared to the number of Chinese workers (see also Cooke 2014 and Tang 2018 for similar findings). In other words, the job creation expectations held by many seemed relatively exaggerated. Moreover, during the construction, increasing awareness of the drawbacks of the approach held by the Chinese contractors—CHEC—developed. The local Ghanaian media was highly sceptical towards the terminal building and the lack of benefits for the local community in particular and Ghana, in general.

Concerning indirect employment effects, MPS officials emphasise that 95% of the materials came from local suppliers. There also seems to be a novel approach to stakeholder management. For example, MPS tried to work against strikes and unrest among stevedore employees, pilots, and various support groups by initiating corporate social responsibility (CSR) activities directed at the local community in Tema town. However, new questions arose: What are the net employment effects of the new MPS terminal, and how will the new terminal influence the long-term employment and human capital composition of employees such as stevedore employees, pilots, and supervisory authorities in the old and the new terminals? MPS estimates that 90% of the port employees who are local can hold better-paying jobs such as modern crane operators, scanner and gate officers, and help desk. These jobs require higher human capital development, but the number of jobs is still uncertain because they depend on future operations and growth. In other words, some actors describe the construction of the port as a success in terms of on-time delivery in agreed-upon quality and better future job opportunities, but not all agree.

## Methodology

In an earlier study, Lawer (2019) tried to map the implication of changes in governance systems in our chosen empirical context. Here, the conclusion is that there are difficulties between the various stakeholders handling complex values and competing interests. The study was published in 2019, hence before the Coronavirus Disease 2019 (COVID-19) pandemic, and we would like to follow up and report on current and further developments after COVID-19.

The paper is based on research conducted by a team of three Danish and two Ghanaian researchers, who were part of the Port Efficiency and Public–Private Capacity at the Port of Tema in Ghana (PEPP) research project funded by the Danish Foreign Ministry and led by Annette Skovsted Hansen. The two rounds of fieldwork carried out in the Spring of 2019 and 2020, respectively, corresponding to approximately six months before and after Terminal 3 opened. The first data collection stretched back to preliminary interviews with officials from the Ministry of Transport, the Ministry of Trade and Industry and the Ghana Ports and Harbours Authority. During the first data collection in Ghana in March and April 2019, before the COVID-19 pandemic and the opening of the new terminal, we met with the following Ghanian authorities: Ghana Ports and Harbours Authority, Ghana Maritime Authority, Ghana Institute of Freight Forwarders (GIFF), Customs Division of the Ghana Revenue Authority, and the local knowledge institutions University of Ghana, Legon, GIFF Education centre and the Regional Maritime University, Nungua.

The first impression of the local stakeholder composition was that many national authorities were involved in Port of Tema’s operations. This seems to differ compared to ports in Europe. In addition, due to the Danish Research Group’s lack of prior knowledge of Ghanaian contextual conditions, the logic was that it is better to include too many stakeholders than too few influential ones. In other words, we took a broadly defined purposeful sampling. Therefore, we organised three focus groups at a kick-off meeting in April 2019 (see Vu and Lützhöft 2020) with 1–2 researchers and 1 secretary in each, with the participation of 54 resource persons related to Port of Tema. They decided which focus group to join. In other words, we assumed that the main interest was the dominant and most efficient allocation principle. The three focus groups were to discuss:

1. Port Efficiency (26 participants)
2. Digitalisation of the port (in general) (20 participants)
3. Capacity development (of local actors) (8 participants)

Job titles included in the focus groups were general managers, directors, senior managers, master mariners, vice presidents, and many middle managers from larger companies. The local Ghana researchers in the group selected the large majority of invited potential stakeholders; conversely, the Danish research group members selected Danish participants from the Danish Embassy and representatives from APM terminals and Maersk. Thus, these stakeholders could provide informative perspectives on port efficiency broadly, both seen from a private and a government perspective. Our aim with selecting a broad range of individuals, rather than focusing on a specific targeted group, such as people directly involved in building the new terminal in Port of Tema, was to gather a wider range of perspectives and knowledge. We felt this was important because we wanted to create information to benefit those influenced by the new terminal. Furthermore, from a research perspective, this more comprehensive information would be more beneficial for us to map new governance principles based on a change in power distribution between various stakeholder groups (public–private, capital labour, local–global, et cetera).

The participants contributed with their immediate knowledge, for example, what they felt were the challenges and practical issues involved in the current operations of the Port of Tema and what they expected of the new terminal. These questions were deliberately relatively open and broad. The main sample questions discussed in groups were the following:

- What is port efficiency/digitalisation/capacity development to you and your company?
- Who are the key stakeholders who influence efficiency/digitalisation/capacity development?
- How can port efficiency/digitalisation/capacity development be improved?
- What are your expectations for port efficiency/digitalisation/capacity development with the new MPS terminal?

**Fig. 3:**
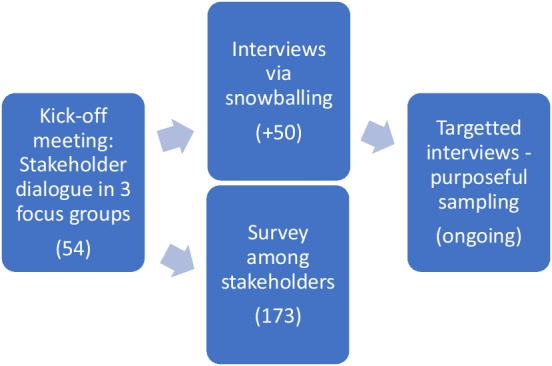
Overview—main methodology elements used in the project

We used focus groups in this initial part of the port extension process, followed by a snowballing method the weeks after the kick-off meeting, to broaden the scope and end up with a more purposeful interview sampling as we gained more knowledge about the field (see Fig. 3). The aim of having three focus groups was to access more in-depth views through dialogue (Bachtin 1981) among individuals. The dialogue flew much freer and allowed participants to convey information we had not expected. In terms of the language used, the interviews were conducted in English and occasionally in Twi, Fante or Akan, when local people were discussing more intensely.

The interconnections between the three themes made it possible to investigate critical questions holistically. For instance, earlier digitalisation attempts were often mentioned as a problem for efficiency, much of which was based on a lack of capacity development. Our fieldwork coincided with the latter part of the actual construction of the new terminal until its first part was operationalised in 2019. That provided a rare opportunity to interact with the key stakeholders and experience the tensions and issues that arose in “real time”. PEPP researchers interviewed while the construction took place, and visited the old port and the new terminal several times.

In the data analysis, a combination of predetermined themes (e.g., objectivist grounded theory) and a continual search for emergent themes (e.g., constructivist) approach was used (Flyvbjerg 2006). In an earlier article, Acheampong et al. (2022) reported on the survey data—investigating the legitimacy of the various stakeholders. In the present article, we have used a higher level of multidisciplinarity. We aimed to gather a wide range of items of theoretical occurrence for consideration by others in their context, gather a body of data for comparison with current research and thinking, and align the data with the sections we had reviewed in the literature. Thus, the analysis was approached more thematically. Specifically, we aim to analyse where there was concurrence with the literature and where participants referred to elements we had not encountered in the maritime literature. This data analysis helped us build a more comprehensive list of items for consideration in the follow-up interviews.

In the study, we adopted a combination of qualitative research methods and a survey to all involved stakeholder organisations with a single respondent reply but measuring relations to other stakeholders. The qualitative case study approach helped us investigate the complex social entity that a port is. To avoid the reported pitfalls of case studies of lack of reliability, validity, and generalisability, we collected data from diverse sources, including state regulatory institutions and private local and foreign-owned businesses, labour and actors in the port city. In addition, we opted to focus our study on stakeholder theory despite its comprehensive coverage in the port economics literature because we had a rare opportunity to follow the implications of different strategic choices while the changes took place, which, in our view, is not reported a lot in the literature.

After the kick-off meeting, the first qualitative data collection took place during March and April 2019 in Accra and in Tema at the old terminals of the Port of Tema and the new MPS Terminal 3, which was under construction. We collected documentary data, interviewed key actors, and visited the Port of Tema, including the Terminal 3 construction site, the Regional Maritime University, Ghana Institute of Freight Forwarders, the Ghana Maritime Authority and Customs Division of the Ghana Revenue Authority and local companies. The second data collection period occurred in February and March 2020 after the Terminal 3 official opening. Below, we have mapped the process based on phase, time-period, main stakeholders, focus and data type (Table 2).

**Table 2:** Qualitative data collection; overview

Phase	Time-period	Main stakeholders	Focus, topics	Type of data
Preparation and building of port Phase I	October 2016–June 2019	CHEC China APM Terminals Ltd and Bolloré Logistics	Delivery on time, with high-quality standards	Historical documents; quantitative data, and post hoc qualitative interviews
Planning, training, technological change etc	November 2017–March 2020	Mapping of training and development initiatives, which groups of employees: port authority, state border agencies and local companies, and partly in concert with Danish specialised companies	Skills, knowledge, and abilities based on the use of modern technologies	Documents and post hoc qualitative interviews
Initial interview round by PEPP’s (Port effectiveness and public private cooperation for competitiveness) research group (what is going on?)	February 2019	Representatives from all actors mentioned in the model	Implications of the new port, efficiency, effectiveness	Ad hoc interviews, snowballing, documents and quantitative data
Start-up of phase I of the new port, inauguration	July 2019	Government, media	Change, speed in capacity use, productivity dip?	Quantitative statistical data
Running of new port	July 2019 = >	Representatives from all actors mentioned in the model	Operations, capacity build-up, use and further development, bottlenecks etc	Structured interviews among key actors; documents and quantitative data analysis on performance

*Source*: Own production

N: + 33 official interviews

Following the thematic content analysis approach, we analysed data from various sources. This approach involved repeatedly consulting the interview transcripts with the recorded sound files and relevant documents to ensure the reliability and validity of the data. First, part of the research team categorised the themes from the data concerning the preparation, planning, construction, start-up and running of the new MPS terminal. Subsequently, we analysed the themes according to the research questions and theoretical framework.

In this initial phase, the multiple stakeholder perspectives offered an opportunity to understand the complexity of the new terminal, not simply as characterised by trade-offs and conflicts. We tried to decode the local system keeping in mind that when we obtained information directly from one source, we had to cross-check by asking for the same information from at least two other informants to avoid information marginalisation. Furthermore, some themes were significantly related to other themes. As the themes were interrelated, our next step was an abstraction discovering the interrelationships of the themes and explaining them using corroborating concepts.

The snowball sampling following the kick-off meeting and focus groups aimed to investigate “what is going on?” Due to the challenges of hard-to-reach populations characterised by a lack of a serviceable sampling frame, the study traced the networks of Ghanaian researchers and their networks combined with Danish researchers’ network in Danish public authorities and the Maersk Group. In such cases, an initial probability sample is either impossible or impractical. Instead, a convenience mechanism determined the initial sample, giving it a nonprobability sample status. Furthermore, in many such hard-to-reach populations as Ghana was for the Danish part of the research team, a link-tracing sampling approach effectively collects data on population members. Finally, the emphasis on tackling reciprocal pre-expectations led us to participate with two representatives in all interviews, i.e., at least one Ghanaian researcher and one Danish researcher.

## Findings

The interview round revealed a shared opinion among the various stakeholders of the importance of increasing the efficiency of the Port of Tema to gain a regional competitive advantage. The new MPS terminal was considered a key element. The size and speed of the change, and not least the technical rationale, which has been emphasised right from the start of MPS’ management, plays a key role in this process. However, local development, understood as a broad array of benefits reaching the local community, like jobs, a steady and increasing income, more business and profit for local companies and not the authorities’ role (and income), has indeed been questioned by many parties. The strong emphasis on efficiency shows that economic reasoning informed the discussion about the expansion of the Port of Tema. The economic rationale is also mirrored in the interviews as much as the discussion centred around the efficiency and effectiveness as a goal accomplishment of the new terminal and the port in general.

In addition, our interviewees seemed to agree that there is a high potential for growth in this part of Africa. They expect shipping freight in and out of Africa to be greater than in the rest of the world in the coming years. In light of this, Ghana is centrally positioned. However, the country itself could not have financed and built the port and achieved as attractive a return as it seems in as much as the International Finance Corporation (IFC), and the two multinational companies—Bolloré and APM Terminals—the total project, leading to legitimate claims of returns on investment of various kinds, from their side. Partly due to the country’s growing debt, which increases financing costs, the foreign investors played a key role and may have been decisive in achieving the high returns already and predicted for the future. According to official documents, the two European multinationals have financed the new MPS terminal. It seems to come down to decision tactics on who benefits most from the investment—pragmatism and political opportunities prevail, compared to strategic, long-term vision-building activities. There is, in other words, a discrepancy between the level of focus and outcome held by the various stakeholders.

APM Terminals and Bolloré, represented by Meridian Port Holdings (MPH) with a combined majority share, seem to engage in strategic decision making to a larger extent. Their focus is on investments and, therefore, on much longer-term returns, whereas the Ghana Government, represented by the public authorities, GPHA, mostly seem to address the tactical level, e.g., interviewees emphasising the importance of political influence, in particular local employment (future voters at elections), and distribution of income among public authorities. Finally, most of the local maritime companies at the port emphasise the difficulties at the operational level before the building of the new terminal and the lack of coordination and dysfunctions of the new systems after the expansion. Many interviewees stressed the difficulties in carrying out their businesses at present and express their hope but also concerns for the future operations after the opening of the new modern terminal. When the existing governance system is not working well, many of the operators are, to a large extent focusing on the operational level, i.e. explaining the dysfunctions of existing systems, and this way, the three main stakeholders tend to meet each other at various analytical levels (see Table 3).

**Table 3:** Main types of stakeholders’ perspective

Stakeholder	Focus	Goal	Dominant fairness-argument
APM terminals and Bolloré logistics	Strategic	Global competitiveness	*We have invested so much money—we need a Return of Investment ROI*
GPHA	Tactic	Continuous income and employment level Regional competitiveness	*We own the port, and we represent the Ghanaian people*
Local private port companies	Operational	System adjustments, local competitiveness	*We want fair competition, then the port will be effective*

*Source*: Own production

In Table 3, we have combined the focus of the three types of stakeholders. We know that all three levels are present in several types of organisations. In trying to understand the strategic options and types of interdependencies between players represented by the terminal operator, the port authorities, and the smaller supply chain stakeholders, we identified what we would call their main focus. When focus levels differ, so do overall goals and the dominant logic of the new port’s purpose. This creates difficulties in meeting the demands of the various stakeholders, which are far more than the three main types mentioned in Table 3. Ports are highly complex hubs with high interdependency among the various stakeholders in the value chain. Cooperation is necessary for the stakeholders to accomplish their goals, so seen from an overall systems perspective, how can they find an appropriate governance model for this particular port that benefits them all?

As mentioned above, a key element in stakeholder theory is the emphasis on norms of fairness in regulating a continuous relationship between stakeholders. In addition, Barney (1986) claims that parties create contracts to coordinate future transactions. However, in many cases, one or both parties inaccurately predict the surplus created through the transaction and seek to renegotiate or even effectively breach the contract when the inaccuracy becomes manifest. This is often a situation where norms of fairness are called upon, followed by renegotiations. In this situation, the advantaged party would handicap their future opportunities by not recognising the roles of fairness norms (see Harrison et al. 2019, p. 7). Still, there are also situations where powerful actors impose new terms on the less powerful transaction partners (e.g., Phillips 2003; Asher et al. 2005).

The various interviewees in and around the Port of Tema and the new MPS terminal tend to emphasise different types of fairness, and it becomes very explicit in the aftermath of the snowballing interview round. When MPS received the concession for Terminal 3 in the Port of Tema, the parties estimated the cost to $1,5 billion. Some parties claim that the cost was lower increasing the return significantly. APM Terminals and Bolloré each had a 35 per cent stake, while GPHA owns 30 percent (Finans 2021). Moreover, the old MPS terminal in Tema, built in 2004, was transferred to GPHA upon completion of the new terminal. Furthermore, a Ministry of Transport of Ghana committee held an enquiry and strongly recommended that the parties renegotiate the contract, to which MPS agreed.

The differences in fairness are further complicated by the latest information about the tender round for the new terminal, which was unexpectedly cancelled by the Ghanaian government (Weir 2021). An article in *African Confidential* (Weir 2021) questions the bidding process for the new MPS terminal in Ghana and criticises the contract between APM Terminals, Bolloré and the Ghanaian Government for being too profitable for the two companies. It is important to note that the journalist who wrote the article in *African Confidential* is from a family who previously had a British shipping line competing with Bolloré and Maersk. However, the Danish newspaper Finans (2021) made similar comments based on their review of the accounts and a confidential excerpt from the Ghanaian Ministry of Transport. The new MPS terminal, which is among Africa’s largest, is expected to yield APM Terminals and Bolloré a return beyond what they normally perform at its terminals (Finans 2021). According to the report, the total port investment of around $1 billion is expected to yield an annual return of 18% over the contract’s life. The return is calculated as the internal interest rate (in comparison, APM Terminals’ total portfolio of terminals delivered a return on invested capital of less than 6 per cent on average in 2017–2020). APM Terminals argues that there is great uncertainty in comparing the returns from the Port of Tema with other investments because local rules and market development can vary greatly—large investments in risky areas require a high return of investment (ROI), or else there are no investments.

In light of this, the outcome of the talks with the many interviewees on the operational level must be seen in a broader political macro-level discussion on *who benefits*, and we might add *how much*, from the new terminal. This way, the various stakeholders’ hear-say, rumours and anecdotes influenced our map. Meanwhile, the new port terminal has more than tripled capacity in the port, and profitability is also likely to improve due to economies of scale. In international container traffic, actors know about the advantages of achieving scale to ensure cost-effectiveness through earnings per container. If earnings keep up with the capacity expansion, it could result in an operating profit of more than $200 million annually. This compares to an investment of around $1 billion to secure the concession for 35 years. So it looks like an excellent business and better than anyone would typically see in the sector (see Finans 2021).

## Conclusion and future developments

Major developments, such as constructing the new MPS terminal, in the Port of Tema, alter the power distribution and rents. Key actors have difficulties finding their roles, gaining transparency to inform their strategic choices and shifting between tactical and strategic levels of operations. Some minor private sector actors tend to stay at the operational level. In contrast, public authorities similarly cling to their tactical-political perspective and the ensuing challenges, such as jobs versus profit and how to design operational systems to benefit all small actors and secure income for government agencies. This leaves strategic considerations and potential advantages to the multinational stakeholders. Still, politics have yet again proven to be the non-predictive factor in the equation, which is common in developing governance models on the African continent.

Despite a systems-level agreement about pursuing goals like growth and effectiveness, aspects like stakeholder fairness based on more pluralist objectives and leading to broader stakeholder inclusiveness seem more difficult to develop. Many political interests are presented, i.e., interviewee answers tend to be strategic, biased and sometimes manipulative, influencing the qualitative data collection process and outcome. It took us a relatively long time to figure out how the economic aspects of the port expansion were composed and, thereby, who benefited most. By describing the changes in the existing structures and systems and assessing the implications for the different actors, we hoped to describe a case that potentially can serve as a model for what to look for when ports are changing governance models, e.g., towards privatisation. What must stakeholders be aware of, and which gaps should they avoid to create a smoother transition?

Several studies have noted the benefits of stakeholder inclusion in planning port infrastructure projects to create mutual sustainability interests or realise shared values (Dooms 2019; Dooms et al. 2013; Parola and Maugeri 2013). Evidence from other studies in recent years has found that stakeholder inclusion and participatory mechanisms have been applied merely as part of a formal procedure in project and infrastructure planning processes to conform to regulatory requirements or to make corporations appear more legitimate (Swyngedouw 2011; Wilson and Swyngedouw 2014). Lawer’s (2019) study of the Port of Tema in the mid-2010 seems to conclude that the inclusive behaviour towards local stakeholders in the Port of Tema takes place at a formal level and serves legitimation purposes. A post-political tool where conflicts emerged due to poor stakeholder management by the authorities, de facto GPHA.

A way out of this is to emphasise the importance of securing the viability of long-term port development plans, which may contribute to sustainable port governance models. This requires stakeholder management capability distributed among the various actors, effective stakeholder communication and consultation and stakeholder involvement in port decisions. In other words, durability and stability in stakeholder relations, stakeholder satisfaction and commitment in the stakeholder relations management process may contribute to port sustainability. However, ensuring that these potential conflicts of interest and external pressures are manageable requires more attention and methods to address the issues (Videira et al. 2012; Vu and Lützhöft 2020; Zaucha and Kreiner 2021). If one is to assume an alignment of the various actor’s perspectives, we have below ordered this in four key processes for stakeholder relations inspired by Godfrey and Lewis (2019, p. 28):

- Leveraging shared values
- Respecting divergent values
- Adoption of an attitude of humility
- Communicate in meaningful ways.

Even though the processes are difficult to contest and, therefore, easily could be labelled “*much more difficult to practice in real life*”, we still consider them meaningful and relevant for future work with the development of port governance models. Despite the various levels of focus, all actors tend, as mentioned above, to share an interest in the effectiveness of the Port of Tema in competition with other local and global ports. Competitiveness means long-term survival, but the income distribution on the way to survival seems to differ. In this respect, it is an example of the classical stakeholder “rights” versus share shareholder value. Here is an example of a West-African logistics infrastructure expansion. This type of conflict has not been solved in many places. In search of a constructive tool, one might look toward social interactions—building on the existing common values—to create peaceable action and co-existence structures. In particular, the latter is essential.

Respect for diverging values mainly looks at the opportunity for the different stakeholders to create protected space for divergent values, e.g., by enacting policies and procedures that recognise and protect the various stakeholders’ fundamental needs and rights. This requires stakeholders to see the limits of their vision and values and accept the validity of others, which could be by admitting mistakes and rectifying stakeholder concerns and issues. For instance, employee safety is the other side of the coin of effectiveness and profit for all the companies in the port.

Finally, meaningful communication can be interpreted in the direction of stakeholders facilitating open dialogue and perhaps formalising communication processes that bring stakeholders and factions of various kinds together, such as local communities, businesses and non-governmental organisations (NGOs). Godfrey and Lewis (2019), from which we have taken most of the contents of the four processes, label this *convergence between stakeholders* based on a preferred and desired moral outcome.

On the practical level, we have tried to add what could be labelled inclusion mechanisms for stakeholders and how these could be enacted through stakeholder engagement. A hopefully more proactive port management approach, where port management needs to consider and integrate the various stakeholders’ concerns and interests into what could be labelled the overall business plan (see also Lawer 2019). Good faith engagement is geared towards mutual gains and not legitimising through formal inclusion without influence (Lawer 2019). Similarly, Lawer emphasises that port management and administrators must ensure that they capture and understand the motives behind their stakeholders’ social, cultural and environmental concerns, interests, and values.

However, the question is whether inclusive port development creates value for all stakeholders. Is it a plus-sum or zero-sum game, where including one part happens at the expense of another’s interests? Unfortunately, many contributions in stakeholder management, also in the empirical studies of the maritime sector, do not address this question—it is, per definition, it is good with a high degree of stakeholder inclusion. However, does it create value for all, and if so, how? If it is a processual measure, probably yes, but if it is based on outcome?

Ports and their stakeholders should consider long-term stakeholder relationships, sharing information and the same corporate philosophy, collaborative spirit, mutual trust, and keeping stakeholders’ common and conflicting interests manageable. Power imbalances in interdependencies—e.g., ports that serve larger and more powerful shipping lines have a lower bargaining power vis-à-vis these parties. They are more strategically dependent on the specific customers who provide the primary input and financial resources.

Theoretically, the case shows that a high level of complexity and various levels of governance systems operating simultaneously necessitate a stakeholder perspective, which can capture the many co-existing realities. One of the strengths of stakeholder theory is the pragmatism and pluralism characterising the approach. At the same time, it offers a moral foundation for shared, sustainable decision-making processes. However, in the present situation and context, a major change in the governance model in a relatively well-organised West African country seems a rather ambitious goal. The main challenge is to find shared norms as a basis for a new governance model when the actors are so diverse, being local–global, capital-labour intensive, small public-large private and exhibiting emphasis on market or tradition for hierarchy.

## Data Availability

The datasets used and/or analysed during the current study are available from the corresponding author on reasonable request.

## Appendix: List of stakeholders

- Ghana Ports and Harbours Authority (GPHA)
- Ghana Maritime Authority (GMA)
- Ghana Institute of Freight Forwarders (GIFF)
- Regional Maritime University (RMU)
- Customs Division of the Ghana Revenue Authority
- Meridian Port Services (MPS)
- Ghana Standards Authority
- Charted Institute of Logistics and Transport (CILT)
- Amaris Terminal
- Chartered Institute of Shipbrokers (ICS), Ghana
- Westblue Consulting
- Ghana Community Network (GCNet)
- Ghana Dock Labour Company
- Customs Division of the Ghana Revenue Authority

## Abbreviations

MPS: Meridian Port Services
IT: Information technology
CHEC: Chinese Harbour and Engineering Company
CSR: Corporate Social Responsibility
GPHA: Ghana Port and Harbour Authority
COVID19: Coronavirus Disease 2019
PEPP: Port Effectiveness and Public Private cooperation for Competitiveness
GIFF: Ghana Institute of Freight Forwarders
IFC: International Finance Corporation
MPH: Meridian Port Holdings
ROI: Return of Investment
